# Impact of tobacco exposure on global deaths of digestive diseases: Findings from 1990–2021 and projected trends to 2042

**DOI:** 10.18332/tid/213468

**Published:** 2025-12-01

**Authors:** Simin Qin, Bowen Xing, Yujun He, Weiai Liu

**Affiliations:** 1 Rehabilitation Medicine Department, The Second Affiliated Hospital of Hainan Medical University, Haikou, China; 2 Preventive Health Center, The First People’s Hospital of Chenzhou, Chenzhou, China; 3 Department of Traditional Chinese Medicine, Taizhou Hospital of Zhejiang Affiliated to Wenzhou Medical University, Taizhou, China; 4 Department of Acupuncture, Tuina and Rehabilitation, Second Affiliated Hospital of Hunan University of Chinese Medicine, Changsha, China

**Keywords:** tobacco exposure, digestive diseases, global burden of disease, predictive analysis, sociodemographic index

## Abstract

**INTRODUCTION:**

Tobacco exposure is a leading modifiable risk factor for preventable deaths, harming the digestive system, and leading to a large number of deaths.

**METHODS:**

A secondary assessment of Global Burden of Disease (GBD) datasets was conducted. From 1990 to 2021, data on deaths from tobacco exposure on digestive diseases, including various genders and age groups, from the 2021 GBD Study were utilized. Trends across 204 countries/territories, 21 GBD, and 5 sociodemographic index (SDI) regions were analyzed. Additionally, a detailed analysis was conducted. Pearson correlation analysis, frontier analysis, age-period-cohort (APC) model, and the Bayesian APC (BAPC) model was utilized.

**RESULTS:**

From 1990 to 2021, the global number of deaths from tobacco-attributable digestive diseases decreased from 52789 (95% uncertainty interval (UI): 36999–68307) to 34061 (95% UI: 23821–46548). The age-standardized rate also decreased, from 1.34 (95% UI: 0.93–1.73) to 0.40 (95% UI: 0.28–0.55). Overall, SDI was negatively associated with disease burden, and the Quality of Care Index for digestive diseases increased with SDI. Results from the APC analysis showed that the mortality rate increased with age; more recently born cohorts had lower mortality rates at the same age; and the mortality rate across all age groups decreased over time. Predictions indicated that the death burden of tobacco-attributable digestive diseases will continue to decline for both males and females from 2022 to 2042.

**CONCLUSIONS:**

Global tobacco-attributable digestive diseases mortality may have decreased due to tobacco control and medical advances. The disease burden in older adults, males, and regions with middle to high SDI deserves special attention. Countries with a relatively high disease burden should learn from those with a lower burden among nations at the same SDI level regarding tobacco control and healthcare management.

## INTRODUCTION

Digestive system diseases represent one of the major global health burdens, encompassing a spectrum of benign and malignant conditions^1^. Data from the Global Burden of Disease (GBD) Study indicate that in 2019, digestive system diseases accounted for approximately 899 million disability-adjusted life years (DALYs) worldwide, representing 3.51% of the total global disease burden and ranking as the 13th leading cause of global health loss^2^. Furthermore, digestive system diseases exhibit distinct disparities across sex, age, and geographical regions, and their pathogenesis is closely associated with multiple risk factors, including smoking, alcohol consumption, high body mass index, and dietary patterns^3^.

Tobacco exposure, encompassing smoking, secondhand smoke, and chewing tobacco, has been identified as one of the leading modifiable risk factors for global preventable deaths. Its health hazards extend beyond the respiratory and cardiovascular systems, exerting broad adverse impacts on the digestive system^4^. A growing body of evidence indicates that harmful constituents in tobacco exert negative effects on the structure and function of multiple digestive organs – either via direct contact or systemic circulation – ultimately inducing inflammation, functional impairment, and even the development of malignant tumors^5^.

Digestive diseases in the GBD 2021 database are classified based on the International Classification of Diseases, 10th Revision (ICD-10). ICD-10 codes relevant to digestive diseases include Chapters K00–K93, encompassing a wide range of lesions from the oral cavity to the rectum^6^. The GBD Study employs a comprehensive approach to estimate disease burden by integrating multi-source data, modeling diseases using the DisMod MR model, and applying a Bayesian statistical framework with Monte Carlo methods to quantify uncertainties. Collectively, these methods form a systematic analytical framework capable of generating comparable and consistent estimates of disease burden^4^. Previous studies based on GBD 2021 have lacked research on the impact of tobacco exposure on overall digestive diseases; for instance, Zi et al.^7^, focused solely on smoking-attributable digestive cancers, and additional studies have focused solely on smoking-attributable peptic ulcers^8,9^. Although all of these studies focused on smoking as a risk factor, smoking is only one form of tobacco exposure – this exposure also includes secondhand smoke and chewing tobacco. By utilizing the GBD framework, this study systematically analyzed and compared the mortality of digestive diseases attributable to tobacco exposure at the global, regional, and national levels.

## METHODS

### Research population and data compilation

This is a secondary dataset analysis of the GBD dataset. The GBD 2021 Study quantified disease burden through a unified analytical pipeline that: 1) harmonized heterogeneous data sources; 2) jointly modeled incidence, prevalence, and mortality within the DisMod MR-2.1 platform; and 3) propagated parameter uncertainty via Bayesian Markov-chain Monte Carlo simulation. This integrated strategy yields internally consistent, cross-epidemiological estimates suitable for global comparison^4^. Studies from the GBD series attach considerable importance to validating the reliability of model-derived results. Specifically, all computations are supplemented with 95% uncertainty intervals (UI); uncertainty aggregation is accomplished by drawing 500 samples from the posterior distribution, with both modeling and sampling uncertainties explicitly incorporated into this process. Such a thorough uncertainty management strategy can alleviate the influence of confounding factors to some degree^10^. The data used in this study were sourced from GBD 2021, and this analysis represents a secondary assessment of datasets derived from the GBD study. Notably, the protocols governing the GBD Study have been extensively detailed in prior research^4^. More specifically, we acquired the following datasets for subsequent analysis: 1) Annual age-specific and age-standardized data on disability linked to digestive diseases (only gallbladder and biliary diseases and upper digestive system diseases were included in the GBD 2021 study) attributable to tobacco, spanning 1990–2021. These indicators encompassed deaths, from which we extracted respective counts, crude rates, and the corresponding age-standardized rates (ASRs). ASRs are calculated using a reference standard population and entail weighted adjustment of incidence rates across individual age groups – a methodological approach that allows for more precise reflection of the actual disparities in disease burden^11^; 2) Global age-stratified population data spanning 1990–2021 were utilized, with the data collection and retrieval period for the project specified as 1 September 2025. Subsequently, we assessed the measured changes across multiple regions between 1990 and 2021, these assessments were conducted for both counts per 100000 population and ASRs; and 3) Regional analyses encompassed 204 countries and territories, which were initially grouped into 21 GBD regions based on geographical proximity and were subsequently categorized into five tiers in accordance with the sociodemographic index (SDI). The SDI is a holistic indicator formulated by researchers involved in the GBD Study, which is designed to evaluate the socioeconomic status of a given region. It incorporates three core dimensions – per capita income, education level, and total fertility rate – into a composite measure normalized to a scale of 0 to 1, which quantifies the socioeconomic vitality and developmental status of a specific region or country. Notably, a higher SDI value reflects enhanced socioeconomic conditions, which are generally linked to superior population health outcomes. For analytical purposes, regions were stratified into five SDI quintiles based on their SDI values: low (0–0.454743), low-middle (0.454743–0.607679), middle (0.607679–0.689504), high-middle (0.689504–0.805129), and high (0.805129–1.0)^12^.

### Data analysis

*Overview*

Data analysis commenced by assessing the structural characteristics of the dataset. Next, we estimated numbers and rates of deaths related to tobacco-attributable digestive diseases, at the global, regional, and national levels. Subsequently, we investigated temporal variations in these metrics across various regions from 1990 to 2021. This comparative analysis focused on two primary outcome measures: the absolute number of cases and age-standardized rates (ASRs) per 100000 population. To quantify long-term temporal trends, the estimated annual percentage change (EAPC) was computed via the formula: EAPC = 100 × (exp(β) - 1), in which β denotes the regression coefficient derived from the linear model. The 95% confidence intervals (CIs) for EAPC were also derived from this linear regression model. Trends were classified according to EAPC and its 95% CI: an upward trend was defined when both EAPC and the lower limit of its 95% CI exceeded 0; a downward trend was identified when both EAPC and the upper limit of its 95% CI were below 0; a stable trend was characterized by the 95% CI of EAPC encompassing 0^13^. Additionally, relative changes in outcome measures between 1990 and 2021 were evaluated via the formula: Relative change (RC) (%) = [(Value in 2021 - Value in 1990) / Value in 1990] × 100%. This computation was applied to both the absolute number of cases and ASRs per 100000 population^13^. All statistical analyses were performed using R software packages (Version 4.2.3). Furthermore, we further analyzed the proportion of deaths from different subtypes among tobacco-attributable digestive diseases, as well as the relationships between disease burden and gender, and between disease burden and age groups. Graphs were generated using R software (Version 4.2.3) and JD_GBDR (Version 2.7.4; Jingding Medical Technology Co., Ltd.).

*Relationship between the burden of tobacco-attributable digestive diseases and SDI*

The association between the death burden of tobacco-attributable digestive diseases and SDI was analyzed using Pearson correlation analysis^12^.

*Relationship between the digestive diseases quality of care index and different SDI levels*

To further elucidate variations in the burden of tobacco-attributable digestive diseases across distinct SDI levels, we examined the association between the digestive diseases Quality of Care Index (QCI) and SDI. Epidemiological indicators employed to quantify the disease’s epidemiological status. Four secondary indicators were additionally computed, each indirectly assessing a specific dimension of digestive diseases care quality: 1) mortality-to-incidence ratio, 2) DALY-to-prevalence ratio, 3) prevalence-to-incidence ratio, and 4) YLLs-to-YLDs ratio (YYR). Principal component analysis was conducted to convert these four newly developed secondary indices into a single aggregated measure termed the QCI; its specific algorithm is detailed in prior publications^14^.

*Frontier analysis for death burden of tobacco-attributable digestive diseases*

To minimize redundancy while upholding methodological rigor, frontier analysis was adopted to quantify the gap between the observed disease burden and its theoretically achievable benchmark. The methodological foundation of the frontier analysis drew on the approach outlined by Guan et al.^15^.

*Age-period-cohort analysis for death burden of tobacco-attributable digestive diseases*

Consistent with GBD classifications, individuals aged under 5 years and over 95 years were grouped into a single category. For APC model fitting, age groups were defined as <5, 5–9, 10–14, ..., ≥95 years, with the ‘0’ group representing the under-five cohort. Data were aggregated into five-year intervals (1992–1996, 1997–2001, ..., 2017–2021) to calculate the total number of cases related to deaths and DALYs across different age cohorts. In the present APC analysis, to resolve the multicollinearity among age, period, and cohort effects, the intrinsic estimator (IE) method was adopted^16^. Models built upon Poisson distributions face an inherent identification challenge: the parameters for age, period, and cohort lack unique estimability without additional constraints, due to the linear dependence among their respective effects. To resolve this issue and yield interpretable outcomes, we incorporated the sum-to-zero constraint – a widely adopted standard in APC modeling – into our analytical framework. Specifically, we constrained the sum of parameter estimates across each dimension (i.e. age groups, time periods, and birth cohorts) to equal zero^17^.

*Predictive analysis for death burden of tobacco-attributable digestive diseases*

To forecast future trends spanning 2022–2042, the BAPC model served as an auxiliary validation method for BAPC-based predictions. The BAPC model is built within the framework of traditional APC models, while integrating Bayesian statistical methods. By leveraging Bayesian techniques, the BAPC model combines prior information with sample data to estimate the posterior distributions of unknown parameters. This approach enables direct approximation of posterior distributions and addresses challenges related to model convergence and mixing, thereby enhancing the reliability and accuracy of the model^18^.

## RESULTS

### Overview of the global burden

*Global and regional trend analysis for death burden of tobacco-attributable digestive diseases*

Globally, the death burden of tobacco-attributable digestive diseases exhibited a significant downward trend between 1990 and 2021. In 1990, the global number of deaths attributable to tobacco-related digestive diseases was 52789 (95% uncertainty interval (UI): 36999–68307), with an ASR of 1.37 per 100000 population (95% UI: 0.93–1.73). By 2021, the number of deaths decreased to 34061 (95% UI: 23821–46545), corresponding to an RC of -35.48%. Concurrently, the ASR dropped to 0.40 per 100000 population (95% UI: 0.28–0.55) with an RC of -70.14%. The EAPC in tobacco-attributable digestive diseases-related death ASR during 1990–2021 was -4.03 (95% CI: -4.10 – -3.97) (Supplementary file Table S1).

Both males and females experienced a downward trend in tobacco-attributable digestive diseases-related death burden, though the burden remained consistently higher in males than in females. In 1990, the number of deaths from tobacco-attributable digestive diseases in males was 45713 (95% UI: 3204–5958), with an ASR of 2.53 per 100000 population (95% UI: 1.75–3.30). By 2021, male deaths decreased to 29226 (95% UI: 20814–39751) (RC: -36.07%), and the ASR fell to 0.75 per 100000 population (95% UI: 0.53–1.02) (RC: -70.37%), with an EAPC of -4.04 (95% CI: -4.10 – -3.98). For females in 1990, the number of deaths from tobacco-attributable digestive diseases was 7076 (95% UI: 4610–9735), with an ASR of 0.35 per 100000 population (95% UI: 0.23–0.48). By 2021, female deaths declined to 4835 (95% UI: 3077–6795) (RC: -31.67%), and the ASR decreased to 0.104 per 100000 population (95% UI: 0.07–0.15) (RC: -69.94%), with an EAPC of -4.04 (95% CI: -4.15 – -3.93) (Supplementary file Table S1).

For regions grouped by SDI, the low-middle SDI region exhibited the largest ASR reduction (RC: -74.43%), whereas the high-middle SDI region had the smallest ASR decline (RC: -61.71%). In terms of the EAPC, the low-middle SDI region showed the fastest decline (EAPC: -4.56; 95% CI: -4.69 – -4.43), while the high-middle SDI region had the slowest (EAPC: -3.30; 95% CI: -3.40 – -3.20). Regarding the reduction in the number of deaths, the high SDI region had the largest decrease (RC: -45.97%), and the high-middle SDI region had the smallest (RC: -24.07%) (Supplementary file Table S1).

Among the 21 GBD regions, Australasia recorded the largest reduction in the number of deaths from tobacco-attributable digestive diseases (RC: -68.50%), while Central Sub-Saharan Africa showed the greatest increase (RC: 40.28%). For ASR reduction, Australasia had the largest decline (RC: -86.74%), and Eastern Europe had the smallest (RC: -3.49%, nearly remaining stable). In terms of EAPC, Australasia exhibited the fastest annual decline (EAPC: -6.24; 95% CI: -6.68 – -5.80), whereas Eastern Europe had the slowest (EAPC: -0.83; 95% CI: -1.18 – -0.47) (Supplementary file Table S1).

Across different age groups, tobacco-attributable digestive diseases-related death burden showed divergent trends: a significant decline in middle-aged and young adults, versus an increase in the elderly. For middle-aged and young adults aged 30–59 years, both the number of deaths and ASR decreased substantially, with the magnitude of reduction slightly narrowing with increasing age. Among these, the 35–39 years age group had the largest reduction in death count (RC: -61.83%) and an ASR RC of -76.03% (EAPC: -4.87; 95% CI: -5.03 – -4.71). By 2021, the number of deaths in this group decreased to 882.06 (95% UI: 608.10–1198.25), with the ASR dropping to 0.16 per 100000 population (95% UI: 0.11–0.21) (Supplementary file Table S1).

In contrast, the number of deaths from tobacco-attributable digestive diseases in the elderly (aged ≥85 years) showed a significant upward trend, with a greater increase in older age subgroups; however, the ASR still decreased (to a less extent than in middle-aged and young adults). Among the elderly, the ≥95 years group had the fastest growth in death count (RC: 111.72%), an ASR RC of -60.45%, and an EAPC of -3.17 (95% CI: -3.31 – -3.04). By 2021, the number of deaths in this group increased to 310.36 (95% UI: 190.31–462.37), with the ASR decreasing to 5.70 per 100000 population (95% UI: 3.49–8.48). Furthermore, the 80–84 years age group had an extremely small reduction in death count (RC: -5.29%) – the only non-elderly group with a near-increase – with an ASR RC of -61.75% and an EAPC of -3.11 (95% CI: -3.18 – -3.03). By 2021, the number of deaths in this group decreased to 3110.64 (95% UI: 2045.40–4388.45), and the ASR dropped to 3.55 per 100000 population (95% UI: 2.34–5.01) (Supplementary file Table S1).

**Figure 1:**
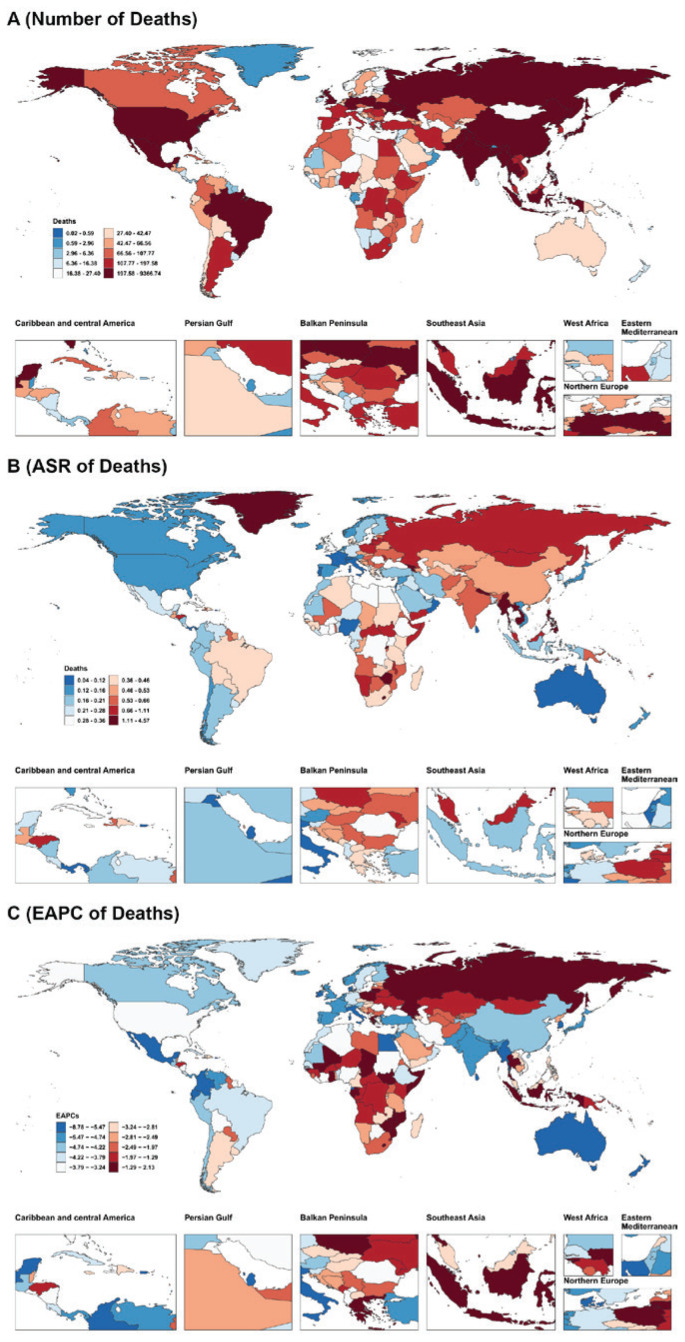
The number (A) and age-standardized rate (B) of deaths from tobacco-attributable digestive diseases in 2021, and the estimated annual percentage change (C) of deaths from tobacco-attributable digestive diseases from 1990 to 2021 across countries (204) and territories worldwide, from Global Burden of Disease 2021 study

The numbers of deaths, along with ASR, for tobacco-attributable digestive diseases in 2021, and EAPC, from 1990 to 2021 across 204 countries and territories globally are shown in Figure 1.

**Figure 2:**
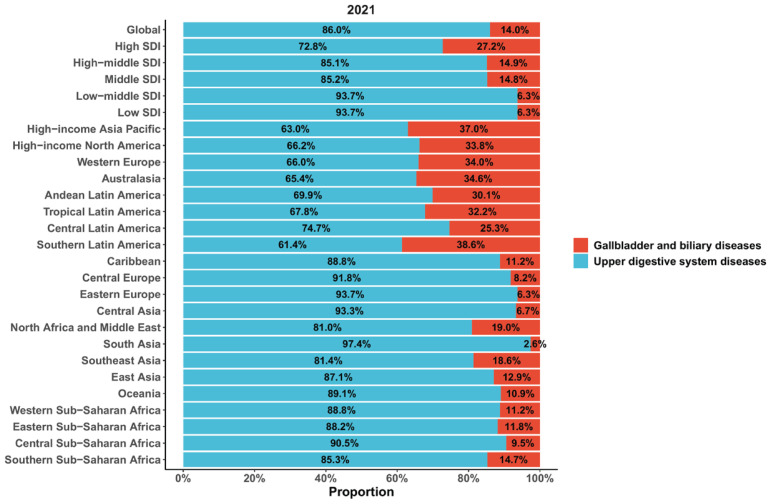
The proportion of each disease among deaths from tobacco-attributable digestive diseases across the global, sociodemographic index regions (5), and Global Burden of Disease regions (21) in 2021, from Global Burden of Disease 2021 study. The shaded area represents the 95% uncertainty interval

In 2021, among global deaths from tobacco-attributable digestive diseases, upper digestive system diseases accounted for 86.0%, while gallbladder and biliary diseases constituted 14.0%. Specifically, the high SDI region had the highest proportion of deaths from tobacco-attributable gallbladder and biliary diseases (27.2%). In contrast, the low-middle SDI and low SDI regions showed the highest proportions of deaths from tobacco-attributable upper digestive system diseases (both 93.7%). Among the 21 Global Burden of Disease regions, South Asia recorded the highest proportion of deaths from tobacco-attributable upper digestive system diseases (97.4%). At the same time, Southern Latin America had the highest proportion of deaths from tobacco-attributable gallbladder and biliary diseases (38.6%) (Figure 2).

*Trends by year, sex, and age for death burden of tobacco-attributable digestive diseases*

Over the past three decades, the absolute number of deaths and the ASR of tobacco-attributable digestive diseases have continued to decrease annually, both globally and across the five SDI regions (Supplementary file Figure S1).

Globally, an analysis of deaths from tobacco-attributable digestive diseases across different age groups and genders revealed characteristic trends. Tobacco-attributable digestive diseases began to cause deaths in humans starting from the 30–34 years age group. The absolute number of deaths increased with increasing age groups until the 65–69 years age group, after which it shifted to a decline. For males, the ASR of deaths from tobacco-attributable digestive diseases showed an upward trend from the 30–34 years age group to the 90–94 years age group, with the upward momentum becoming increasingly pronounced as the age group advanced; this trend then turned to a decline in the ≥95 years age group. In contrast, the ASR of deaths from tobacco-attributable digestive diseases in females exhibited a consistent upward trend across all age groups (Supplementary file Figure S2).

### Relationship between for death burden of tobacco-attributable digestive diseases and SDI

Globally, the SDI was significantly associated with the ASR of deaths caused by tobacco-attributable digestive diseases (r= -0.4973; 95% CI: -0.5646 – -0.4319), p<0.0001). The result is non-linear. Notably, the disease burden exhibited phase-specific changes with increasing SDI. Beginning at an SDI of 0, the disease burden increased with rising SDI; subsequently, an inflection point emerged at an SDI of approximately 0.45, after which the disease burden decreased as SDI continued to rise. Thereafter, a second inflection point appeared at an SDI of around 0.65, and the disease burden resumed an upward trend with increasing SDI. Then, a new inflection point occurred at an SDI of roughly 0.7, following which the disease burden decreased markedly with further increases in SDI (Supplementary file Figure S3A). Among the 204 countries and regions worldwide, this pattern was nearly consistent (Supplementary file Figure S3B).

### Relationship between the digestive diseases QCI and different SDI levels

Results of the QCI analysis for digestive diseases showed that, overall, the QCI was higher in regions with a high SDI and lower in regions with a low SDI. Furthermore, the high-middle SDI and middle SDI regions, as well as the low-middle SDI and Low SDI regions, exhibited partial overlap – indicating that the QCI levels among these regions were not completely distinct (Supplementary file Figure S4).

### Frontier analysis for death burden of tobacco-attributable digestive diseases

Frontier analysis further revealed the impact of the SDI on the ASR of tobacco-attributable digestive diseases. As shown in the left panel of Supplementary file Figure S4, nearly all countries saw their disease burden gradually move closer to the frontier line between 1990 and 2021, and this trend was consistent regardless of their SDI levels.

Notably, among low SDI countries, Somalia was closest to the frontier line; among low-middle SDI countries, Niger and Burkina Faso were also close; and among middle SDI countries, Benin and Liberia were closest. However, most countries far from the frontier line also belonged to the middle SDI region, such as Cambodia and Kiribati. Among high SDI and high-middle SDI regions, countries including the United Kingdom and the Republic of Korea were closer to the frontier line, while countries like Greenland and Lithuania remained further from it (Supplementary file Figure S5).

### APC analysis for death burden of tobacco-attributable digestive diseases

Results of the APC analysis showed the following:

- Age effect: A consistent age-dependent increase was observed across all periods. The death rate rose significantly from the young adult group (30–34 years: 0.10–0.33 per 100000 population) to the elderly group (≥95 years: 6.10–13.68 per 100000 population) (Supplementary file Figure S6A).
- Cohort effect: Within the same birth cohort, the death rate increased with age. However, more recent birth cohorts had lower death rates at the same age (Supplementary file Figure S6B).
- Period effect: Within the same age group, the death rate decreased significantly over time. This pattern was observed across all age groups (Supplementary file Figure S6C).

### Predictive analysis for death burden of tobacco-attributable digestive diseases

The BAPC-based prediction results indicated that, given the year-on-year decrease in the death burden of tobacco-attributable digestive diseases from 1990 to 2021, this burden will continue to decline over the next 20 years (2022–2042), with this downward trend observed in both males and females (Supplementary file Figure S7).

## DISCUSSION

### Global and regional trend analysis

Between 1990 and 2021, the global number of deaths from tobacco-attributable digestive diseases decreased. Concurrently, the ASR also declined. The EAPC indicates a stable and significant year-on-year downward trend in mortality risk. This trend is closely associated with the global promotion of tobacco control policies, increased awareness of tobacco-related harms, and advancements in medical care, as elaborated below: From the perspective of tobacco control policies, since the World Health Organization Framework Convention on Tobacco Control (WHO FCTC) came into force in 2005, over 180 countries worldwide have implemented measures such as tobacco tax increases, smoke-free environment legislation, tobacco packaging warnings, and the popularization of smoking cessation services^19^. These interventions directly led to a reduction in the global level. For instance, globally, the tobacco control policies (2007–2014) cumulatively averted 22 million smoking-related deaths, with tobacco tax policies making the largest contribution (averting 7 million deaths)^20^. Reduced tobacco exposure can significantly decrease the accumulation of carcinogens (e.g. nicotine, benzo[a]pyrene) in the digestive mucosa, thereby lowering the incidence and mortality risks of tobacco-attributable digestive diseases such as esophageal cancer, gastric cancer, and liver cancer^4^. From the perspective of medical interventions, over the past three decades, significant advancements have been made in early diagnostic technologies and treatment methods for digestive diseases. Particularly in high-income regions, these advancements have effectively reduced the death rate of tobacco-attributable digestive diseases, further amplifying the mitigating effect of tobacco control on the death burden.

The death burden of tobacco-attributable digestive diseases in males has consistently been higher than that in females. However, the reduction magnitude in the number of male deaths was slightly greater than that in females, while the reductions in ASR and the EAPC were highly comparable between the two genders. The core reason for this discrepancy lies in gender differences in tobacco exposure history. Historically, the smoking rate among males has been significantly higher than that among females^21^. This difference is not only reflected in smoking rates but also extends to smoking behavior, health impacts, and smoking cessation success rates^21^, leading to a higher initial death burden of tobacco-attributable digestive diseases in males.

Among the 21 GBD regions, Australasia exhibited the most favorable outcomes regarding tobacco-attributable digestive diseases. Since the 1970s, Australia has significantly reduced smoking rates through a series of innovative policies, such as substantial increases in tobacco taxes, the implementation of plain tobacco packaging, bans on tobacco advertising, and the promotion of smoke-free environments^22^. In stark contrast were Middle Sub-Saharan Africa and Eastern Europe, Countries in Central Sub-Saharan Africa have generally ratified the WHO FCTC; however, policy implementation is hindered by insufficient alignment, industry interference, and intersectoral fragmentation. For instance, the tobacco economy remains a key source of revenue in some countries (e.g. Malawi, Zambia)^23^, while demand-side measures such as tobacco tax policies and advertising bans suffer from inadequate enforcement^24^. Meanwhile, economic regulations are often prioritized by non-health sectors, which conflicts with tobacco control objectives^23^. In Eastern Europe (e.g. Russia, Ukraine), smoking is deeply entrenched in the culture, and the tobacco industry wields strong influence^25^.

The age-related disparity in the death burden of tobacco-attributable digestive diseases is the most prominent. The burden decreased significantly among middle-aged and young adults aged 30–59 years, whereas the number of deaths increased among the elderly aged ≥85 years. This pattern reflects the combined effects of the ‘intergenerational effect of tobacco exposure’ and ‘population aging’. For middle-aged and young adults aged 30–59 years, particularly the 35–39 years age group, their growth period (after 2000) coincided with the intensive implementation of global tobacco control policies, resulting in low levels of tobacco exposure^19^. This highlights the characteristic of tobacco control policies – ‘earlier intervention yields earlier benefits’. For the elderly population aged ≥85 years, although the ASR still decreased, the number of deaths rose significantly. The core reasons are twofold: first, high intergenerational tobacco exposure – this population was in their youth during the mid-20th century, when global smoking rates were at their peak^26^, and the accumulated risk of tobacco-attributable digestive diseases manifested concentratedly in old age; second, intensified population aging – the growth in the population base amplified the upward trend in the number of deaths. For the 80–84 years age group, the number of deaths showed an RC of -5.29% (nearly stable). This is likely because their tobacco exposure history coincided with the ‘pre-tobacco-control peak’, leading to a relatively high accumulated risk.

In 2021, among global deaths from tobacco-attributable digestive diseases, upper digestive system diseases accounted for as high as 86.0%, while gallbladder and biliary diseases accounted for only 14.0%. This compositional difference stems from variations in the strength of association between tobacco and different digestive system diseases. The mucosa of the upper digestive system is directly exposed to tobacco combustion products or tobacco components ingested orally^27^. Carcinogens in tobacco can directly damage the DNA of mucosal epithelial cells, induce gene mutations, and trigger abnormal cell proliferation, which further progresses to precancerous lesions or even carcinogenesis^5,28^.

Although carcinogens in tobacco can reach the biliary system via the bloodstream, their concentration is far lower than that in the mucosa of the upper digestive system, resulting in a lower probability of inducing carcinogenesis. Carcinogens in tobacco (e.g. nitrosamines, polycyclic aromatic hydrocarbons) may promote biliary carcinogenesis by directly damaging bile duct epithelial cells or inducing chronic inflammatory responses^29^. Furthermore, components in tobacco smoke may exacerbate biliary diseases via oxidative stress and immunosuppression^30^.

### Synthesis of gender and age trends

Global deaths from tobacco-attributable digestive diseases exhibited distinct characteristics by age and gender. Deaths from this disease begin in the 30–34 years age group, as the effects of tobacco on the digestive system require time to accumulate. The number of deaths peaks in the 65–69 years age group – when cumulative tobacco-induced damage is most severe and the body’s repair capacity is compromised – after which it declines. This post-peak decline occurs because individuals aged ≥70 years are more prone to dying from other conditions such as heart disease and pneumonia, which reduces the overall number of deaths from tobacco-attributable digestive diseases. In terms of gender differences, for males, the mortality risk in the 30–94 years of age increases rapidly with age, driven by higher smoking prevalence and longer smoking duration that lead to faster accumulation of tobacco-related damage. After 95 years of age, the risk may decline, as other age-related diseases are prioritized as the cause of death. For females, likely primarily due to exposure to secondhand smoke, the mortality risk continues to rise across all age groups.

### Relationships between SDI and death burden of tobacco-attributable digestive diseases

The QCI for digestive diseases shows a gradual upward trend with increasing SDI, which can explain, to some extent, the inflection points in the death burden of tobacco-attributable digestive diseases as SDI changes. This is because there is partial QCI overlap between high-middle SDI and middle SDI regions, as well as between low-middle SDI and low SDI regions. This study revealed that the SDI is significantly negatively associated with the death burden of tobacco-attributable digestive diseases, and it exhibits distinct phased changes with increasing SDI. When SDI <0.45, in low SDI regions, increased tobacco accessibility coupled with insufficient medical resources leads to a rise in death burden as SDI increases^31^. For the 0.45–0.65 SDI phase, socioeconomic development facilitates the implementation of tobacco control policies and advancements in medical technology^32^, resulting in a subsequent decline in death burden. For the 0.65–0.7 SDI phase, in high-middle SDI regions, synergistic risks may arise from factors such as population aging and increased dietary risks^33^. Combined with delayed improvements in healthcare quality, this leads to a short-term rebound in death burden. When SDI ≥0.7, in high SDI regions, the combined effects of strict tobacco control and precision medicine result in a significant reduction in death burden.

### APC analysis of the death burden of tobacco-related digestive diseases

The age, period, and cohort effects revealed by the APC analysis in this study provide a valuable perspective for understanding the dynamic characteristics of the death burden of tobacco-related digestive diseases:

- Age effect: The mortality rate showed a significant age-dependent increase with advancing age, with the mortality rate differing by more than 60-fold between the 30–34 years age group and the ≥95 years age group. This is consistent with the cumulative nature of tobacco harm and the pathological mechanisms of the disease. Carcinogens in tobacco (e.g. benzo[a]pyrene) require long-term exposure to induce malignant transformation of digestive mucosal cells^34^. Additionally, the elderly exhibit declining digestive function and reduced immune clearance capacity, which further exacerbates the risk of disease-related death.
- Cohort effect: More recently born cohorts had lower mortality rates at the same age, reflecting improvements in intergenerational tobacco exposure patterns. With the global popularization of tobacco control policies (e.g. youth tobacco prohibition regulations, tobacco harm awareness campaigns), recent cohorts have experienced lower tobacco exposure during their growth stages.
- Period effect: The mortality rate decreased significantly over time across all age groups, which is directly associated with advancements in public health and medical care during the same period. On one hand, the promotion of the WHO FCTC has reduced the overall population’s tobacco consumption rate^35^; on the other hand, the widespread adoption of early diagnostic technologies for digestive diseases (e.g. endoscopic screening) and anti-tumor treatments (e.g. targeted drugs) has effectively lowered the disease-related mortality rate across all age groups – demonstrating the overall effectiveness of cross-age-group interventions.

### Prevention and control strategies for death burden of tobacco-attributable digestive diseases under frontier analysis

From 1990 to 2021, nearly all countries moved closer to the frontier line in terms of the death burden of tobacco-attributable digestive diseases, regardless of their SDI levels. This confirms the synergistic effect of global tobacco control efforts and advancements in medical care.

Most countries far from the frontier line are classified as middle SDI countries – a pattern that highlights the role of SDI in shaping the death burden of tobacco-attributable digestive diseases. High SDI countries are typically characterized by robust healthcare systems and effective tobacco control policies, enabling them to achieve lower mortality rates compared to countries with other SDI levels. This reflects successful management of disease burden. In contrast, countries far from the frontier line – particularly many middle SDI countries – may face challenges such as limited healthcare infrastructure and suboptimal tobacco control measures, resulting in higher than expected mortality rates.

Furthermore, analysis revealed that SDI levels and distance from the frontier line are not linearly associated. Although global efforts to control disease burden have achieved significant results, significant disparities in disease burden persist across countries. Countries with severe disease burden should learn from countries with similar SDI levels that are closer to the frontier line, including: low SDI countries (e.g. Somalia); low-middle SDI countries (e.g. Niger, Burkina Faso); middle SDI countries (e.g. Benin, Liberia); high SDI countries (e.g. the United Kingdom); and high-middle SDI countries (e.g. South Korea). In contrast, Greenland and Lithuania are far from the frontier line and require further development and refinement of public health policies.

### Predictive analysis of death burden of tobacco-attributable digestive diseases and discussion on prevention and control strategies

Based on the predictive results of the BAPC model, the death burden of tobacco-attributable digestive diseases will continue the annual downward trend observed from 1990 to 2021 through 2022–2042, with both male and female populations benefiting synchronously. This conclusion provides forward-looking confidence for global tobacco control and digestive disease management.

The core support for this predicted trend lies in the sustained effectiveness of tobacco control policies (e.g. the implementation of the WHO FCTC) and medical technologies (e.g. early diagnosis and treatment of digestive diseases) over the past three decades. These two types of interventions have formed a stable health gain mechanism, whose long-term impacts are sufficient to sustain the burden reduction over the next 20 years. The consistency of trends between males and females also suggests that existing interventions are not gender-biased, with universal coverage and implementation effectiveness.

### Limitations

This study has certain limitations. First, limitations related to data dependence and heterogeneity. This study relies on secondary data from the GBD 2021 and although it covers a wide range of regions, there are variations in data collection methods, quality, and completeness across countries/regions. Low SDI regions, due to inadequate medical recording systems, may have biases in tobacco exposure assessment or missing mortality data for digestive diseases. This leads to insufficient representativeness of results in these regions and may result in underestimating or overestimating the actual disease burden. Second, predictions from the BAPC model are based on the assumption that current tobacco control intensity and healthcare standards remain unchanged. The model does not account for potential impacts on disease burden from future factors such as loosening of tobacco control policies, popularization of emerging tobacco products, or public health emergencies^36^. This may result in deviations between the predicted results for 2022 to 2042 and actual outcomes. Third, in the frontier analysis, the assumption of optimized management in frontier countries fails to consider the influence of genetic susceptibility or colonial history on healthcare systems and tobacco control policies. Thus, localized validation of the proposed policies is required. Furthermore, in population-level GBD data, residual confounding factors may exist due to unmeasured individual factors (e.g. smoking intensity), making it difficult to establish a causal relationship. As an ecological analysis, it carries the risk of ecological fallacy when inferring individual-level associations; additionally, multiple comparisons can lead to the inflation of Type I errors. Finally, this type of study is unlikely to establish a causal relationship; additionally, the studies conducted in the specific locations included in the discussion do not necessarily exhibit generalizability.

### Future research

For future research, it is recommended to consider dose-response curves between tobacco exposure and specific diseases. Point-to-point quantitative results will facilitate a more accurate assessment of the burden attributable to tobacco exposure. Therefore, given the spatiotemporal variations in tobacco exposure sources and the susceptibility characteristics of specific populations, it is scientifically more reasonable to compare the tobacco exposure-attributable burden across different populations in different regions. Finally, tobacco exposure not only causes deaths from digestive diseases but also leads to disability. However, the GBD database currently lacks data on disability-related outcomes for many regions. Future studies could conduct further analyses once this data gap is addressed. Nevertheless, despite these limitations, the results of this study provide valuable epidemiological evidence and offer targeted interventions for countries worldwide, aiming to alleviate the burden of tobacco exposure and its associated digestive diseases.

## CONCLUSIONS

From 1990 to 2021, the global death burden of tobacco-attributable digestive diseases decreased significantly. Males bore a heavier death burden, yet their mortality rate showed a slight decline; in contrast, the mortality rate among individuals aged ≥85 years continued to rise. Notably, upper digestive system diseases are the major cause of global deaths from tobacco-attributable digestive diseases. Predictions from the BAPC model indicate that the mortality rate of tobacco-attributable digestive diseases will continue to decline for both males and females from 2022 to 2042. Despite this positive outlook, priority should still be given to focusing on the elderly population and high-middle SDI regions. Maintaining tobacco control efforts, strengthening disease screening, and monitoring emerging tobacco risks are key to further alleviating the burden of tobacco-attributable digestive diseases.

## Supplementary Material



## Data Availability

The data supporting this research are available from the following link: https://vizhub.healthdata.org/gbd-results/
