# Supplementary material for: Impact of tobacco exposure on global deaths of digestive diseases: Findings from 1990–2021 and projected trends to 2042
Source: Tob Induc Dis. 2025 Dec 1;23:10.18332/tid/213468. doi: 10.18332/tid/213468 (PMC12670142; doi:10.18332/tid/213468)
Supplement: Supplementary file 1 [file TID-23-187-s1.pdf]

Table S1 The case number and ASR of deaths from tobacco-attributable digestive diseases in 1990 and 2021, and its temporal trends from 1990 to 2021, categorized by global, SDI and 21 regions. (Data Source: Global Burden of Disease Study 2021, Covering 204 Countries and Territories)

Location name/sex/ Age group	1990		2021		1990-2021		
	**Case number (95% UI)**	**ASR per 100,000 (95% UI)**	**Case number (95% UI)**	**ASR per 100,000 (95% UI)**	**EAPC % (95% CI)**	**RC of numbers (%)**	**RC of ASR (%)**
Global	52789(36999-68307)	1.34(0.93-1.73)	34061(23821-46548)	0.40(0.28-0.55)	-4.03(-4.10,-3.97)	-35.48	-70.14
**Sex**							
Male	45713(32037-59578)	2.53(1.75-3.30)	29226(20814-39751)	0.75(0.53-1.02)	-4.04(-4.10,-3.98)	-36.07	-70.37
Female	7076(4610-9735)	0.35(0.23-0.48)	4835(3078-6795)	0.10(0.07-0.15)	-4.04(-4.15,-3.93)	-31.67	-69.94
**SDI**							
High SDI	7954(5481-10507)	0.72(0.50-0.95)	4298(2872-5933)	0.20(0.14-0.28)	-4.28(-4.52,-4.04)	-45.97	-71.86
High-middle SDI	10180(7156-13186)	1.04(0.73-1.35)	7730(5411-10381)	0.40(0.28-0.53)	-3.30(-3.40,-3.20)	-24.07	-61.71
Middle SDI	15765(11009-20946)	1.57(1.08-2.09)	10920(7536-15246)	0.43(0.29-0.60)	-4.19(-4.28,-4.10)	-30.73	-72.8
Low-middle SDI	14843(9922	2.34(1.55-3.	8382(5563-	0.60(0.39-0.	-4.56(-4.68,-4.4	-43.53	-74.4

	-20740)	30)	11871)	85)	3)		3
Low SDI	3996(2603-5435)	1.72(1.12-2.34)	2695(1741-3821)	0.53(0.34-0.74)	-3.98(-4.13,-3.83)	-32.57	-69.17
**Regions**							
Andean Latin America	168(111-240)	0.84(0.55-1.19)	120(73-180)	0.20(0.12-0.31)	-4.69(-4.88,-4.50)	-28.72	-75.57
Australasia	144(96-193)	0.61(0.41-0.82)	45(29-66)	0.08(0.05-0.12)	-6.24(-6.68,-5.79)	-68.5	-86.74
Caribbean	254(173-345)	0.98(0.67-1.33)	190(124-266)	0.35(0.23-0.49)	-3.56(-3.79,-3.34)	-25.19	-64.11
Central Asia	404(286-508)	0.81(0.57-1.02)	484(341-652)	0.56(0.39-0.75)	-1.92(-2.22,-1.61)	19.66	-30.95
Central Europe	1747(1246-2229)	1.18(0.84-1.51)	1278(877-1699)	0.62(0.43-0.82)	-2.20(-2.49,-1.91)	-26.87	-47.46
Central Latin America	875(620-1140)	1.12(0.78-1.46)	656(437-901)	0.27(0.18-0.37)	-5.06(-5.29,-4.82)	-25.05	-76.16
Central Sub-Saharan Africa	169(102-268)	0.73(0.44-1.16)	238(136-368)	0.40(0.23-0.62)	-1.72(-1.88,-1.56)	40.28	-45.41
East Asia	15720(10878-21030)	1.94(1.34-2.61)	9663(6283-14601)	0.47(0.30-0.71)	-4.41(-4.56,-4.26)	-38.53	-75.75
Eastern Europe	2433(1809-3042)	0.87(0.65-1.	2776(2010-3573)	0.84(0.61-1.	-0.83(-1.18,-0.4	14.1	-3.49

		09)		08)	7)		
Eastern Sub-Saharan Africa	938(523-1370)	1.13(0.65-1.64)	1023(621-1530)	0.52(0.33-0.77)	-2.52(-2.55,-2.48)	8.99	-54.01
High-income Asia Pacific	1387(966-1805)	0.73(0.51-0.95)	764(493-1070)	0.14(0.10-0.20)	-5.49(-5.66,-5.32)	-44.88	-80.26
High-income North America	1536(1033-2104)	0.43(0.29-0.59)	932(598-1306)	0.14(0.09-0.20)	-3.80(-4.18,-3.42)	-39.36	-66.81
North Africa and Middle East	1448(942-1983)	0.89(0.58-1.25)	1094(710-1544)	0.26(0.17-0.37)	-4.32(-4.53,-4.10)	-24.4	-70.54
Oceania	50(32-70)	1.62(1.07-2.28)	65(42-95)	0.80(0.52-1.18)	-2.38(-2.45,-2.31)	30.35	-50.35
South Asia	14950(9963-21265)	2.51(1.63-3.58)	7337(4490-10784)	0.52(0.31-0.77)	-5.22(-5.38,-5.05)	-50.92	-79.29
Southeast Asia	4161(2677-5997)	1.62(1.05-2.33)	3659(2521-5037)	0.58(0.40-0.80)	-3.50(-3.65,-3.36)	-12.06	-64.18
Southern Latin America	290(200-386)	0.62(0.43-0.84)	169(111-233)	0.20(0.13-0.27)	-3.49(-4.01,-2.98)	-41.71	-68.37
Southern Sub-Saharan Africa	261(180-358)	0.96(0.65-1.33)	306(204-418)	0.51(0.34-0.70)	-1.77(-2.12,-1.42)	17.52	-46.57
Tropical Latin America	1017(718-1315)	1.12(0.78-1.46)	1005(658-1408)	0.39(0.26-0.55)	-3.84(-4.16,-3.52)	-1.25	-65

Western Europe	4382(3012-5796)	0.75(0.52-0.99)	1742(1124-2427)	0.18(0.12-0.24)	-4.85(-5.04,-4.66)	-60.25	-76.55
Western Sub-Saharan Africa	455(301-634)	0.49(0.32-0.70)	515(330-719)	0.24(0.15-0.33)	-2.37(-2.54,-2.21)	13.37	-51.45
**Age group**							
30-34 years	1353(963-1766)	0.35(0.25-0.46)	550(384-746)	0.09(0.06-0.12)	-4.642(-4.805,-4.477)	-59.353	-74.083
35-39 years	2311(1650-3014)	0.66(0.47-0.86)	882(609-1198)	0.16(0.11-0.21)	-4.870(-5.032,-4.708)	-61.826	-76.025
40-44 years	3040(2191-3884)	1.06(0.76-1.36)	1302(923-1757)	0.26(0.18-0.35)	-4.970(-5.141,-4.799)	-57.169	-75.472
45-49 years	3737(2669-4849)	1.61(1.15-2.09)	1820(1263-2415)	0.38(0.27-0.51)	-4.824(-4.936,-4.711)	-51.301	-76.119
50-54 years	5169(3707-6666)	2.43(1.74-3.14)	2576(1796-3460)	0.58(0.40-0.78)	-4.855(-4.941,-4.768)	-50.17	-76.193
55-59 years	6209(4368-8027)	3.35(2.36-4.33)	3507(2468-4756)	0.89(0.62-1.20)	-4.362(-4.480,-4.243)	-43.516	-73.566
60-64 years	6832(4845-8778)	4.25(3.02-5.47)	3821(2684-5157)	1.19(0.84-1.61)	-4.308(-4.432,-4.184)	-44.076	-71.936
65-69 years	6903(4799-9031)	5.58(3.88-7.31)	4416(3061-5986)	1.60(1.11-2.17)	-4.178(-4.271,-4.086)	-36.036	-71.337

70-74 years	6017(4062-7934)	7.11(4.80-9.37)	4349(3072-6008)	2.11(1.49-2.92)	-3.963(-4.037,-3.889)	-27.724	-70.273
75-79 years	5243(3500-6959)	8.52(5.69-11.31)	3867(2639-5297)	2.93(2.00-4.02)	-3.451(-3.516,-3.385)	-26.242	-65.574
80-84 years	3284(2193-4461)	9.28(6.20-12.61)	3111(2045-4388)	3.55(2.34-5.01)	-3.105(-3.178,-3.032)	-5.288	-61.745
85-89 years	1900(1243-2613)	12.57(8.23-17.30)	2415(1569-3432)	5.28(3.43-7.51)	-2.871(-3.020,-2.721)	27.131	-57.983
90-94 years	644(413-906)	15.04(9.64-21.14)	1135(735-1641)	6.35(4.11-9.17)	-2.837(-2.922,-2.752)	76.196	-57.794
95+ years	147(92-212)	14.40(9.08-20.86)	310(190-462)	5.69(3.49-8.48)	-3.174(-3.305,-3.042)	111.716	-60.453

Note: regions were stratified into five SDI quintiles based on their SDI values: low (0–0.454743), lower-middle (0.454743–0.607679), middle (0.607679–0.689504), upper-middle (0.689504–0.805129), and high (0.805129–1). UIs are for GBD outputs, and CIs are for model-based estimates.

Abbreviations: ASR, age-standardized rate; RC, relative change; EAPC, estimated annual percentage change; SDI, sociodemographic index; UI, uncertainty interval; CI, confidence interval.

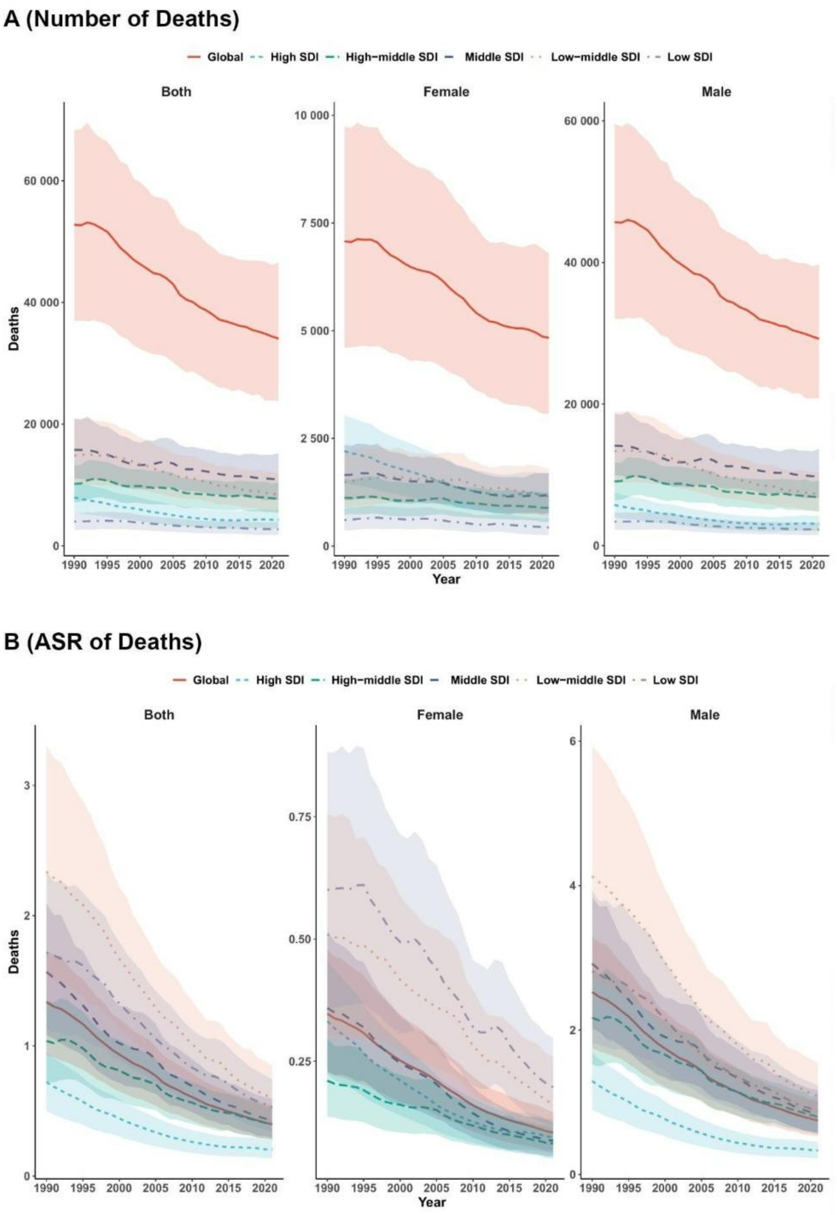

Figure S1 The global and Socio-demographic Index regions (5) trends of number (A) and age-standardized rate (B) in deaths from tobacco-attributable digestive diseases from 1990 to 2021 from Global Burden of Disease 2021 study.

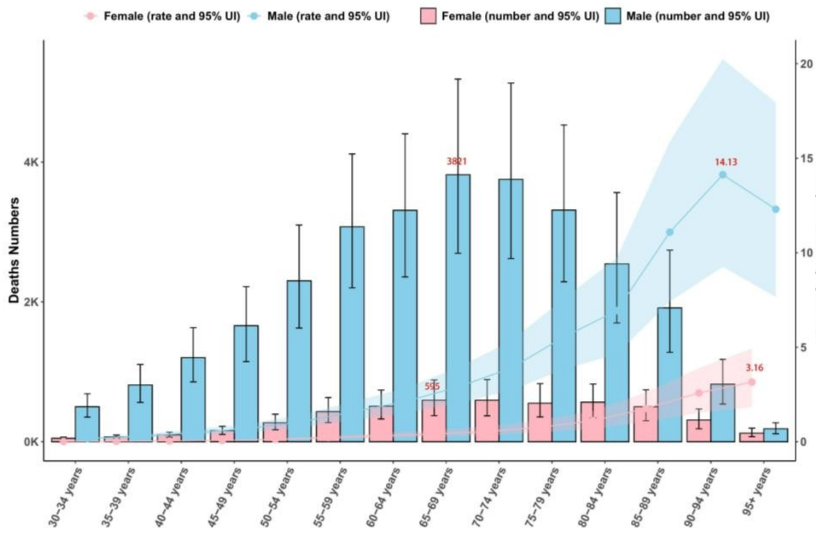

Figure S2 The trends of case number and age-standardized rate in deaths from tobacco-attributable digestive diseases across different genders, female and male, by age groups in global in 2021 from Global Burden of Disease 2021 study. The red font represents the maximum value. Bar represents number and the trend line represents rate. The shaded areas are 95% uncertainty interval (UI).

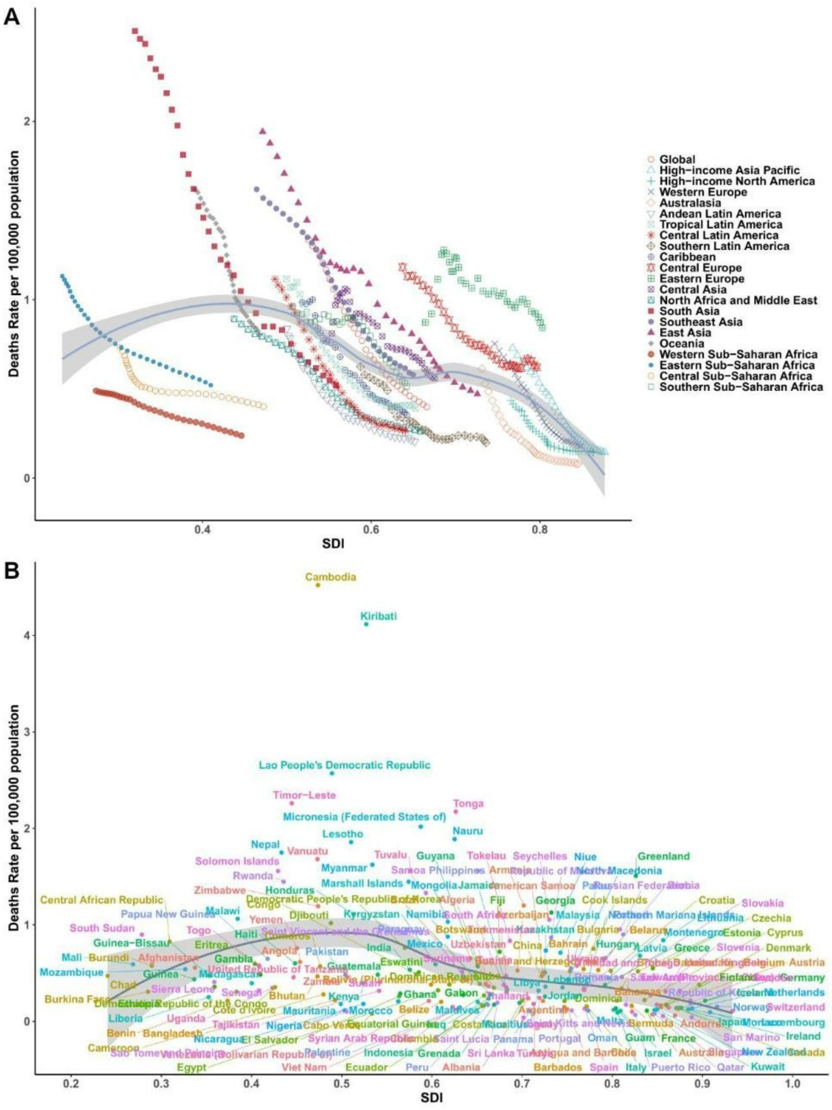

Figure S3 Relationship between Socio-demographic Index and the deaths from tobacco-attributable digestive diseases: results across Global Burden of Disease regions (21) (A) and countries and territories (204) (B) in 2021 from Global Burden of Disease 2021 study. The shaded regions represent the predicted trend and its 95% confidence interval.

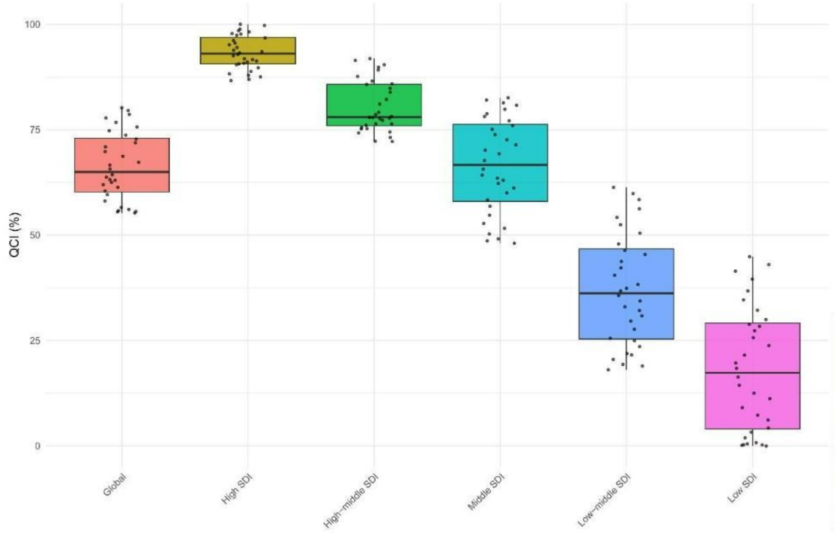

Figure S4 Comparison of the Quality of Care Index (QCI) across global and different Socio-demographic Index regions (5) for digestive diseases in 2021 from Global Burden of Disease 2021 study. Colored box: The lower edge of the box corresponds to the 25th percentile (Q_1) of QCI; The upper edge of the box corresponds to the 75th percentile (Q_3) of QCI; The box covers the "middle 50%" range of QCI data in this group. Black horizontal line inside the box: represents the median of QCI in this group. Scatter points (small black dots): each scatter point represents the raw QCI value of an individual sample in this SDI group.

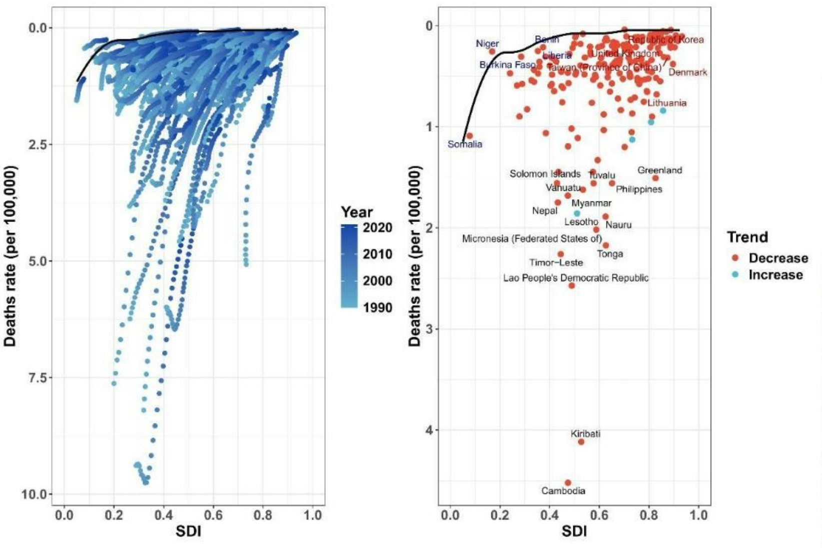

Figure S5 Frontier analysis, represented by the solid black lines, explores the relationship between Socio-demographic Index (SDI) and age-standardized rate for deaths in the context of tobacco-attributable digestive diseases in countries and territories (204) from Global Burden of Disease 2021 study. The color gradient in graphs A illustrates the progression of years, ranging from light shades representing 1990 to the darkest shades denoting 2021. In graphs B, each dot signifies a specific country or territory for the year 2021, with the top countries (15) displaying the most significant deviation from the frontier labeled in black. Countries with low SDI (> 0.455) and minimal deviation from the frontier are highlighted in blue, while those with high SDI (> 0.805) and notable deviation for their developmental level are emphasized in red. The direction of change from 1990 to 2021 in age-standardized rate is indicated by the color of the dots: red dots represent decrease, while blue dots signify increase.

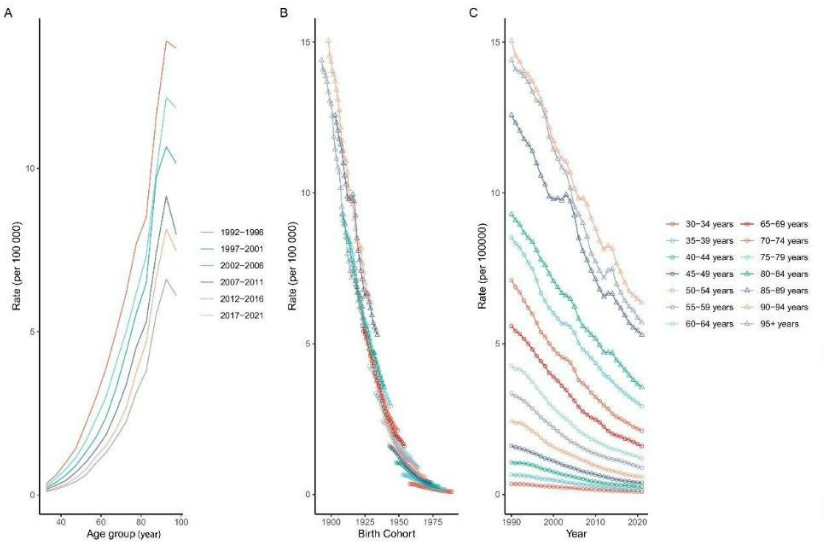

Figure S6 Results of the APC analysis from 1990-2021 for deaths from tobacco-attributable digestive diseases from Global Burden of Disease 2021 study. A: Age effect; B: Cohort effect; C: Period effect.

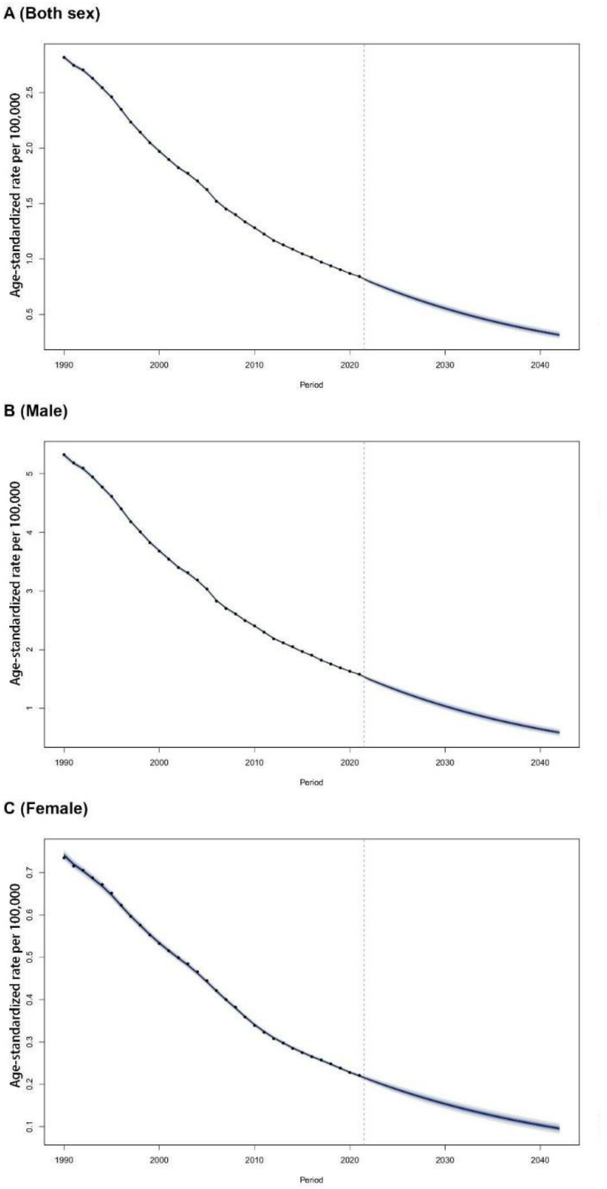

Figure S7 Predicted trends of tobacco-attributable digestive diseases over the next 20 years (2022-2042) (A: both sex; B: male; C: female) from Global Burden of Disease 2021 study. Dark blue lines with dots represent the true trend during 1990-2021; Dark blue lines without dots and shaded regions represent the predicted trend and its 95% confidence interval.

© 2025 Qin S. et al.
